# Protective role of Mannose binding Lectin (MBL2) promoter haplotypes on TB infection in South Indian HIV-1 patients

**DOI:** 10.1186/1471-2334-14-S3-P37

**Published:** 2014-05-27

**Authors:** Kalaimani Pandian, Stalinraja Maruthamuthu, Suresh Madasamy, Jayalakshmi Mariakuttikan

**Affiliations:** 1 Department of Immunology, School of Biological Sciences, Madurai Kamaraj University, Madurai- 625021, India; 2 ART Centre, Govt. Theni Medical College and Hospital, Theni- 625512, India

## Background

Mannose binding Lectin (MBL) mediates protection against infections by activating the complement system, but certain microorganisms may increase infectivity by exploiting this host defence system. Hence, the purpose of this study is to evaluate MBL genetic variants with the development of TB infection caused by *Mycobacterium tuberculosis* among HIV patients.

## Methods

Blood samples from TB+ART+ and TB^-^ART+ (n=30) were collected. Genomic DNA was extracted from Peripheral Blood Mononuclear Cells (PBMC) using salting out procedure. MBL promoter haplotypes of -550 H/L and -221Y/X associated with high, medium and low (HY, LY and LX) secretion was assessed by PCR-SSP.

## Results

The Promoter haplotype (LY/LX) associated with deficient MBL levels conferred a protective role to TB in our study population with a significant difference (Chi-square (*X^2^*) =4.00; p<0.05).

## Conclusion

In this study, we could observe MBL2 promoter haplotypes with low MBL secretion may play a protective role to intracellular mycobacterium infections like TB in HIV seropositive individuals.

